# Interventions and adaptations to strengthen data quality and use for COVID-19 vaccination: a mixed methods evaluation

**DOI:** 10.1093/oodh/oqae010

**Published:** 2024-05-06

**Authors:** Godefroid Mpanya, Constant Kingongo, Julia Ngomba, Erick Biduaya Panu, Papy Mbokolo, Djeneba Coulibaly, Sang Dao Dinh, Dung Tham Chi, Trung Pham, Thao Le, Hawa Idde, Yannick Agui, Wendy Prosser, Ana Costache, Audry Hong, Elan Ebeling, Grace Awantang, Jessica C Shearer

**Affiliations:** MOMENTUM Routine Immunization Transformation and Equity, PATH, 119, Blvd du 30 Juin, Immeuble Matrix, 5e niveau, Commune de la Gombe, Kinshasa, DRC; MOMENTUM Routine Immunization Transformation and Equity, PATH, 119, Blvd du 30 Juin, Immeuble Matrix, 5e niveau, Commune de la Gombe, Kinshasa, DRC; MOMENTUM Routine Immunization Transformation and Equity, PATH, 119, Blvd du 30 Juin, Immeuble Matrix, 5e niveau, Commune de la Gombe, Kinshasa, DRC; Consultant, PATH, Kinshasa, DRC; Consultant, PATH, Kinshasa, DRC; MOMENTUM Routine Immunization Transformation and Equity, PATH, 119, Blvd du 30 Juin, Immeuble Matrix, 5e niveau, Commune de la Gombe, Kinshasa, DRC; MOMENTUM Routine Immunization Transformation and Equity, PATH, R11.01, 11th Floor, Hanoi Towers, 49 Hai Ba Trung, Hoan Kiem District, Hanoi, Vietnam; MOMENTUM Routine Immunization Transformation and Equity, PATH, R11.01, 11th Floor, Hanoi Towers, 49 Hai Ba Trung, Hoan Kiem District, Hanoi, Vietnam; MOMENTUM Routine Immunization Transformation and Equity, PATH, R11.01, 11th Floor, Hanoi Towers, 49 Hai Ba Trung, Hoan Kiem District, Hanoi, Vietnam; MOMENTUM Routine Immunization Transformation and Equity, PATH, R11.01, 11th Floor, Hanoi Towers, 49 Hai Ba Trung, Hoan Kiem District, Hanoi, Vietnam; MOMENTUM Routine Immunization Transformation and Equity, JSI, Niamey, Niger; MOMENTUM Routine Immunization Transformation and Equity, JSI, Niamey, Niger; MOMENTUM Routine Immunization Transformation and Equity, JSI, Arlington, JSI, 2733 Crystal Drive, Arlington, VA 22202, USA; MOMENTUM Routine Immunization Transformation and Equity, JSI, Arlington, JSI, 2733 Crystal Drive, Arlington, VA 22202, USA; MOMENTUM Routine Immunization Transformation and Equity, PATH, 2201 Westlake Avenue, Seattle, WA 98121, USA; MOMENTUM Routine Immunization Transformation and Equity, PATH, 2201 Westlake Avenue, Seattle, WA 98121, USA; MOMENTUM Routine Immunization Transformation and Equity, JSI, Arlington, JSI, 2733 Crystal Drive, Arlington, VA 22202, USA; MOMENTUM Routine Immunization Transformation and Equity, PATH, 455 Massachusetts Ave NW, Washington, DC 20001, Washington, DC, USA

**Keywords:** immunization, COVID-19, health management information system, data use, adaptive management, data quality

## Abstract

Many countries used digital health solutions to support COVID-19 vaccination but struggled to implement them, resulting in adaptations. This theory-driven mixed methods evaluation of COVID-19 vaccine-related data and digital interventions from the Democratic Republic of the Congo, Niger and Vietnam aimed to uncover (i) what drove mid-course adaptations of these digital health interventions, (ii) how these adapted interventions may have contributed to improved availability, quality and use of COVID-19 vaccine-related data and (iii) if and how these interventions strengthened eHealth building blocks. Methods consisted of interviews, document review, secondary data analysis and observation. Findings indicated that decisions to adapt original interventions were driven by need and the availability of funding. Adapted interventions improved the availability and quality of data. Data use improved in all three countries although there were ongoing challenges observed in the Democratic Republic of the Congo and Niger. The interventions did not appear to strengthen the eHealth building blocks, although in the Democratic Republic of the Congo and Niger they had positive effects on routine immunization systems. Achieving longer-term improvements in eHealth building blocks requires intentional focus from the design stage, which may be more challenging in an emergency context.

Abrégé

De nombreux pays ont utilisé des solutions de santé numériques pour appuyer la vaccination contre la COVID-19, mais ont eu du mal à les mettre en œuvre, ce qui a conduit à des adaptations. Cette évaluation à base théorique des données liées au vaccin contre la COVID-19 et des interventions numériques de la République démocratique du Congo, du Niger et du Vietnam vise à découvrir (1) ce qui a motivé les adaptations à mi-parcours de ces interventions de santé numérique, (2) comment ces interventions adaptées ont pu contribuer à accroître la disponibilité, la qualité, et l’utilisation des données relatives au vaccin contre la COVID-19 et (3) si et comment ces interventions ont renforcé les composantes de cybersanté. Les méthodes ont compris des entretiens, un examen des documents, une analyse des données secondaires et l’observation. Les résultats indiquent que les décisions d’adapter les interventions originales étaient dictées par les besoins et la disponibilité des financements. Les interventions adaptées ont permis d’accroître la disponibilité et la qualité des données. L’utilisation des données s’est améliorée dans ces trois pays, bien que des problèmes persistent en République démocratique du Congo et au Niger. Les interventions n’ont pas semblé renforcer les composantes de cybersanté, bien qu’elles aient eu des effets positifs sur les systèmes de vaccination systématique en République démocratique du Congo et au Niger. Améliorer les composantes de cybersanté nationales sur le plus long terme exige une intentionnalité accrue dès la phase de conception, ce qui peut être plus difficile dans un contexte de crise.

Resumen

Muchos países utilizaron soluciones de salud digital para apoyar la vacunación contra la COVID-19, pero tuvieron dificultades para implementarlas, lo que dio lugar a adaptaciones. Esta evaluación de los datos relacionados con la vacuna contra la COVID-19 y las intervenciones digitales realizadas en la República Democrática del Congo, Níger y Vietnam, basada en la teoría y en métodos mixtos, tiene como objetivo descubrir: (1) qué impulsó las adaptaciones de estas intervenciones de salud digital a medio camino, (2) cómo estas intervenciones adaptadas pueden haber contribuido a mejorar la disponibilidad, la calidad y el uso de los datos relacionados con la vacuna contra la COVID-19, y (3) si estas intervenciones fortalecieron los componentes básicos de la cibersalud y cómo lo hicieron. Los métodos consistieron en entrevistas, revisión de documentos, análisis de datos secundarios y observación. Los hallazgos indicaron que las decisiones de adaptar las intervenciones originales fueron impulsadas por la necesidad y la disponibilidad de fondos. Las intervenciones adaptadas mejoraron la disponibilidad y la calidad de los datos. El uso de datos mejoró en los tres países, aunque se siguieron observando problemas en la República Democrática del Congo y Níger. Las intervenciones no parecieron fortalecer los componentes básicos de la cibersalud, si bien en la República Democrática del Congo y Níger tuvieron efectos positivos en los sistemas de vacunación sistemática. Para lograr mejoras a largo plazo en los componentes básicos de la cibersalud se requiere un enfoque deliberado desde la etapa de diseño, lo que puede resultar más difícil en un contexto de emergencia.

## INTRODUCTION

Reaching priority populations with COVID-19 vaccines and achieving vaccine equity require that public health authorities have access to and can use reliable and timely data. Strengthening the use of information in immunization programs requires that relevant data are available when needed, and that health workers, managers and their teams have the skills, opportunities and motivation to review, discuss and take programmatic action [1, 2].

Digital health interventions (DHIs) are digital technologies that have a discrete functionality related to health sector objectives [3]. Adoption and use of DHIs in a nonemergency context can take years of careful planning and change management [4–10]. The unprecedented context of the COVID-19 pandemic and rapid development of the COVID-19 vaccine created an urgency to vaccinate as many people as quickly as possible and led to the rapid adoption or development of vaccine data-related DHIs. The concepts of *rushed deployment* and *rushed adoption* of technologies during COVID-19 are not unique to vaccination data systems; periods of crisis spur rapid innovation generally [4]. However, many vaccine data-related DHIs adopted rapidly by national governments with minimal consideration of readiness faced barriers to uptake and use, resulting in suboptimal availability and use of data for vaccination planning and decision-making.

In efforts to improve these DHIs, ministries of health (MOHs) in the Democratic Republic of the Congo (DRC), Niger and Vietnam set out to adapt DHIs or their implementation to address challenges and gaps in DHIs’ software, hardware, workflows and change management processes, resulting in adapted interventions. We define *interventions* to broadly encompass the entire suite of interventions targeting a specific programmatic outcome. We define *adoption* as the initial decision to use a new intervention and *adaptation* as the set of revisions and changes to improve the intervention. The adapted interventions may look very different from the original interventions adopted, but they typically seek to achieve the same goals. The adaptation processes in the three countries led to the following interventions: revised immunization information systems and tools to improve data quality and data use in DRC; digital remote monitoring systems in Niger for vaccine temperature control and revised microplanning systems and processes in Vietnam. The MOMENTUM Routine Immunization Transformation and Equity project (the project) supported these efforts. The project is a 5-year award funded by the U.S. Agency for International Development (USAID) to increase equitable immunization coverage in USAID-supported countries around the globe. The evaluation described in this article explored several questions regarding to what extent and how adaptations of DHIs were successful. Specific evaluation questions were as follows:

1. How were COVID-19 vaccine-related data and digital interventions refined or adapted based on gaps and challenges identified? To what extent, and why or why not, did adaptations consider the countries’ digital health strategies or architecture or leverage existing global goods?
2. Whether and how did these adaptations or new strategies contribute to improved outcomes related to the availability, quality and use of COVID-19 vaccine-related data for program decision-making?
3. Whether and how did these digital health adaptations or new strategies strengthen the digital health enabling environment across the eHealth building blocks [11] in evaluation countries or the broader health or immunization system? What were the barriers and facilitators to strengthening the digital health enabling environment or health systems through these investments?

## METHODS

### Evaluation setting and interventions

We purposively selected country cases that reflected different levels of digital health maturity and a range of intervention types to optimize potential for learning. The following sections provide contextual information on each of these countries and the intervention context. Further information on the interventions is presented in Table 1 and in the results section for the first evaluation question, which explored how and why these adaptations occurred.

**Table 1:** Interventions evaluated

**Country**	**Specific interventions evaluated**
DRC	• Provision of financial support to ensure the availability of paper forms at supported health facilities and paying data entry staff to retroactively enter historical vaccination data from paper forms into the DHIS2 aggregate module • Provision of training and supportive supervision of health zone staff to enter data into the DHIS2 aggregate module and of site-level staff to use the revised paper COVID-19 data entry tools that facilitate DHIS2 aggregate data entry • Provision of technical assistance for monthly data review meetings at the health zone level • Funding for back entry of historical vaccination data from paper forms into the DHIS2 aggregate module • Collaboration with the EPI to update forms and DHIS2 aggregate modules to include new disaggregation of vulnerable populations (refugees and internally displaced people, people with disabilities and people living in prisons)
Niger	• Support for the adoption of a digital remote temperature monitoring device • Development and adaptation of standard operating procedures for collection, use of temperature data and ensuring preventive maintenance • Capacity strengthening activities (training and supportive supervision) for cold chain users and technicians to look at, interpret and take action on dashboard temperature data • Provision of guidance to incorporate temperature and maintenance data into monthly government regional reports and ongoing MOH data reviews at the national level
Vietnam	• Development of an easy-to-use Excel-based microplanning tool for improved planning, based on a World Health Organization tool • Addition of automated stock forecasting functionality based on the number of COVID-19 vaccine doses needed for each commune, based on the population data entered in the tool • Development of operational guidelines to improve adoption and correct use of the tool and the microplanning process • Training and supportive supervision to support adoption and correct use of the tool and the microplanning process

#### Democratic Republic of the Congo

At the time of writing this paper (May 2023), 96 652 cases of COVID-19 had been confirmed in DRC. The COVID-19 vaccine was rolled out in April 2021, and as of May 2023, 15.5% of the population had received at least one dose of the vaccine [12]. In 2019, DRC launched an agency for digital health and developed a national health informatics strategy in 2020, which identified the District Health Information Software 2 (DHIS2) to manage and report aggregate data for most routine health services including immunization. In 2021, DRC adopted DHIS2’s COVID-19 Tracker module, an electronic immunization registry to collect and monitor individual-level COVID-19 vaccination records with the functionality to produce digital vaccine certificates [13].

Although DHIS2 COVID-19 Tracker aligned with the country’s existing digital health architecture, its implementation faced many challenges touching on most aspects of the eHealth building blocks. These included unavailability of paper forms at vaccination sites needed to prepare data for data entry, unavailability of tablets necessary for accessing the module, delays in generating user logins for the DHIS2 Tracker module, inadequate internet and electricity, insufficient human resources to enter client data, insufficient training and supervision and lack of payment of staff leading to low motivation, capacity and opportunity to use Tracker [14]. Relatively early in the COVID-19 vaccination response, the government introduced a parallel Excel database to address the data entry backlogs and missing data resulting from these implementation challenges; however, this resulted in multiple workflows and the lack of a single source of data. In September 2022, the Expanded Programme on Immunization (EPI) reported that 13.6 million vaccine doses had been administered based on vaccine stock data. Of these estimated doses delivered, only 27% were reported in the Excel database and less than 10% of the total doses estimated to be administered were reported in DHIS2 Tracker. The large discrepancy between the stock data and the records of vaccines administered reflected, in large part, a significant delay in the flow of vaccination data from paper records at vaccination sites to the national information systems. Evidence of these backlogs motivated national partners, with the project’s support, to identify and implement system and process improvements.

#### Niger

As of May 2023, 9513 cases of COVID-19 had been reported and 23.7% of the Nigerien population had received at least one dose of the COVID-19 vaccine [15]. A low-resource country in the Sahel, Niger is more prone to vaccine cold chain disruptions than most other countries. A 2019 assessment found that at the national level, 36% of vaccine cold chain equipment (CCE) reported temperatures that were outside the appropriate temperature range, threatening vaccine potency [16]. Niger, like many other countries, used a device called a 30-Day Temperature Logger to track temperatures of CCE. These devices are placed in the CCE and collect temperature data for up to 60 days; however, they require manual data download and analysis by health workers instead of remote and automated monitoring of data by cold chain managers and technicians. The manual nature of the system made it challenging to identify temperature excursions—readings outside the recommended range—in real time. Temperature excursions result in decreased vaccine potency. As COVID-19 vaccines were being introduced in March 2021, the Ministry of Public Health, Population and Social Affairs (MOH) EPI logistics team recognized the need for improved temperature monitoring of the CCE.

In 2021, the project supported the MOH to partner with Parsyl, Inc. to pilot digital remote temperature monitoring devices (RTMDs) in two regions (Dosso and Tillaberi) to assess the feasibility of using them. RTMDs monitor the temperature of CCE, send text message alerts to health workers for immediate action when there is a temperature excursion and send real-time data to a dashboard. Following the pilot, by September 2022 devices (donated by Parsyl) were scaled up to additional regions and the district level, with technical support provided by the project. The availability of funding, combined with the political priority of improving the cold chain for COVID-19 vaccines, contributed to this decision.

#### Vietnam

By May 2023, 91% of Vietnam’s population had received at least one dose of a COVID-19 vaccine [17] but health staff and managers faced challenges with microplanning for the remaining unvaccinated communities and individuals. Microplanning is the process health workers and managers use to identify priority communities and develop workplans for vaccination services. In August 2021, the MOH developed a COVID-19 vaccination microplanning module within their national immunization information system; however, the module had several limitations including personal identification requirements that excluded some clients, particularly undocumented populations; limited functionality for estimating vaccine stocks; limitations on the timeline for registering subjects; and the requirement for internet connectivity, which was a challenge in remote communes and districts. To fill the gap, immunization managers at the district and commune levels rapidly developed ad hoc tools using Microsoft Word and/or Excel for their own microplanning efforts. These tools varied widely in design, complexity and the data elements included, but they helped to overcome the limitations of the national microplanning module. However, most of these ad hoc tools required manual data calculations, contributing to data quality issues. The MOH required an updated microplan every time national target populations changed, meaning that significant human resource time was spent on manually updating microplans. An assessment in Vietnam co-led by the project, the National Institute of Hygiene and Epidemiology and the Pasteur Institute, identified the COVID-19 microplanning process as a particular challenge for health workers in the five project provinces. To address these challenges, the project supported the adaptation and implementation of the World Health Organization’s (WHO’s) Excel-based microplanning tool for COVID-19 vaccination [18] in Vietnam in February 2022 at the provincial, district and commune levels in two provinces (Dien Bien and Son La) and in March 2022 in three additional provinces (Hoa Binh, Quang Nam and Ninh Thuan). The resulting Excel-based tool was developed in collaboration with the national EPI, regional EPIs and provincial centers for disease control. The project included complementary activities to improve the roll-out and use of the new microplanning tool.

**Table 2:** Geographic regions sampled

Evaluation country	Geographic regions sampled
DRC	Two health zones (Matete and Limete) in Kinshasa province; 12 health facilities
Niger	Four districts (Aguie, Tessaoua, Illela and Bouza) in 3 regions (Tahua, Maradi and Niamey)
Vietnam	Four districts (Lac Thuy, Kim Boi, Nam Gian and Dong Giang) in two provinces (Hoa Binh and Quang Nam); 16 communes

### Evaluation design

We conducted a multicountry theory-informed mixed methods process and outcomes evaluation to assess why interventions were adapted and their resulting outcomes. Considering the complexity of the interventions and implementation contexts, we drew on complexity-aware monitoring approaches [19], including causal link monitoring [20] and outcome harvesting [21] to inform interview guides and analysis approaches. We undertook this evaluation as part of a set of learning activities across four global USAID-funded projects [22].

### Evaluation theory of change

USAID and the four projects implementing the learning activities jointly developed a theory of change (TOC) to describe the hypothesized relationships between COVID-19 vaccine-related digital and data investments and improved outcomes and impacts for COVID-19 and the health system [22, Figure 1]. The TOC informed our overall evaluation questions, methods, data collection tools and analysis approaches; e.g. our second evaluation question assessed the TOC’s immediate and intermediate outcomes, including data availability, data quality and the use of data for vaccination program planning. To understand why these changes occurred—or not—we collected data to measure constructs related to implementation [23] and changes in users’ capabilities, motivation and opportunities to use the interventions or their data [24].

The third evaluation question explored the intermediate outcomes at the digital health ecosystem and health system level, including the effects of the interventions on the eHealth building blocks of leadership and governance; strategy and investment; services and applications; standards and interoperability; infrastructure; legislation, policy and compliance; and workforce [11]. We also sought to understand whether these interventions—primarily designed to address short-term needs to support COVID-19 vaccine uptake—have broader consequences on the broader immunization or health system. Due to the differences in interventions and contexts, not all aspects of the TOC were measured in all countries.

The first evaluation question—how and why were COVID-19 vaccine-related data and digital interventions refined or adapted?—is not directly reflected in the TOC. We conceptualized it as a step prior to the ‘interventions’ outlined in the TOC; a process of technical and political negotiation resulting in the selection of interventions. We drew on policy science frameworks [25, 26] to inform our interview guides and data analysis for this question.

### Evaluation population and sampling strategy

Administrative areas and key informants were selected using a combination of convenience and purposive sampling to obtain a variety of perspectives on COVID-19 vaccination data systems at several levels of the health system. All countries selected respondents from the national and second and third administrative levels (e.g. provinces and districts). In DRC, the evaluators selected health workers at health facilities. Table 2 summarizes the geographic regions included in each country’s evaluation.

### Data collection

Multidisciplinary teams, including project monitoring and evaluation staff and independent evaluators, collected data through in-depth interviews, document review, secondary data analysis and observation between April and June 2023. Table 3 summarizes how each data collection approach was applied to answer the evaluation questions in each country.

**Table 3:** Description of evaluation methods by type and country (corresponding evaluation questions in parentheses)

**Type of data collection activity**	**DRC**	**Niger**	**Vietnam**
Interviews	Semistructured interviews with key informants from frontline health facility staff, health system managers and national stakeholders to understand the how and why of adaptations (1) and to assess improvements in data quality, data availability and data use (2) as well as changes to eHealth building blocks (3)	Semistructured interviews with key informants to understand how and why the RTMD intervention was introduced (1) Interviews with national immunization supply chain staff, cold chain technicians, and health facility staff to understand changes in capacity, motivation and opportunity to use RTMD data (2), changes in data availability and use (2) and changes to eHealth building blocks (3)	Semi-structured interviews with key informants to understand the how and why of adaptations (1). Interviews with provincial and district health officials and commune-level health workers responsible for microplanning to understand whether adaptations improved capacity, motivation and opportunity for microplanning (2), as well as data use, data quality and data availability (2) the microplanning process (2) and changes to eHealth building blocks (3)
Document review	Project reports and workshop slides to triangulate the timing of adaptations and re-construct the process of adaptation (1)	Review of monthly regional logistics reports incorporating temperature data to assess data availability and use (2)	N/A
Secondary data analysis	Analysis of available data in DHIS2 Tracker and DHIS2 aggregate to assess data quality with an emphasis on completeness of data (2)	Review of report data synthesizing human-centered design (HCD) interviews to assess the CCE maintenance system in Niger, particularly the challenges and opportunities it presents, including temperature monitoring for decision making (2) Review of Parsyl RTMD dashboard showing CCE temperature data to assess that data’s availability and whether actions were logged (data use) (2)	Analysis of health facility microplan data preintervention and postintervention to assess data availability and completeness (2)
Observations	Observation of health facility worker data management skills and their perceived norms to assess data management capacity (1, 2, 3) Observation of health zone data review meetings to assess data use (2)	Observation of immunization supply chain logisticians discussing temperature data from RTMDs during regional and national data review meetings to assess data use (2)	N/A

#### Interviews

Evaluation teams in all countries used semistructured interview guides to conduct in-depth interviews with technical and financial partners and health systems managers and staff involved in the COVID-19 vaccination effort. In DRC and Vietnam, this included those responsible for vaccinating clients and recording, managing or using vaccination data. In Niger, this included cold chain and logistics staff and managers responsible for managing the vaccine cold chain system. Interviewers asked generally about any observed changes or outcomes to allow for the collection of unanticipated phenomena. Interviewers took notes of all interviews and recorded interviews when they had permission. In Niger, the team also reanalyzed interview data available from a recent human-centered design (HCD) research report (forthcoming) related to the vaccine cold chain.

#### Document review

DRC and Niger teams reviewed project documents, meeting minutes and presentations, national or subnational plans, strategies, operational guidelines and reports to assess how and why adaptations occurred. The Niger evaluators reviewed monthly regional logistics reports from two regions to determine if temperature data were integrated into those reports.

#### Secondary data

Evaluation teams gathered existing quantitative data to assess changes in data availability, quality and use. The DRC team compared the total number of vaccination doses reported in the DHIS2 aggregate and Tracker systems for the two evaluation health zones from April 2021 to June 2023 to assess data availability and completeness. In Niger, the evaluation team reviewed the Parsyl dashboard and a Parsyl-generated report to assess whether RTMDs were sending data and making it available and whether target staff logged any actions taken. In Vietnam, the evaluation team reviewed 22 preintervention and postintervention microplans in the sites visited against 22 indicators required for the development of a high-quality microplan—e.g. whether the microplan included an estimate of the target population size—to assess changes in data availability for these indicators.

**Table 4:** Summary of data collection, by country

**Type of data collection activity**	**DRC**	**Niger**	**Vietnam**
In-depth key informant interviews	15 individual interviews (5 national; 2 antenna^a^; 4 health zone; 2 facility)	8 individual interviews (4 regional; 4 district); HCD synthesis report (synthesized information from 23 in-depth interviews)	30 individual interviews (4 provincial; 8 district; 17 commune; 1 project staff)
Secondary data analysis	Review of DHIS2 and Excel data	Review of Parsyl dashboard and reports and monthly regional logistics reports incorporating temperature data	Review of 22 microplans
Observations	2 health zone data review meetings; 11 facility visits	2 data review meetings at the national level	N/A

^a^In DRC, the ‘antenna’ level of the immunization program is responsible for immunization functions in the provincial health office.

#### Observation

In DRC, the team observed local monthly health zone meetings and visited health facilities to document data use and data management practices, respectively. During health zone meetings, the team used a semistructured and tailored tool adapted from the PRISM Routine Health Information System checklist [27] to record data relating to the meeting’s agenda, who was present or absent, the quality of the displayed or discussed data, the engagement of participants and the use of data and external factors (e.g. poor internet connectivity) that may have impacted the discussion. A second observation tool was used at the facility level to assess the availability of hard copy paper tools, perceived clarity of staff’s data-related roles, the capacity of staff to enter data and the frequency of data review at the facility. In Niger, the team observed national and regional data review meetings to assess whether temperature data were discussed and used for programmatic action. No observations were conducted in Vietnam.

### Data preparation and analysis

In DRC and Vietnam, interview notes were imported into qualitative data analysis software (Dedoose version 9.0.46); in Niger, interview notes were summarized in a coding table. Teams applied deductive codes based on the evaluation questions that helped explain processes of change and intended outcomes. Teams developed additional inductive codes based on emergent key themes. Teams analyzed data by writing and discussing summaries of key codes to identify patterns. Description of key themes across excerpts, and any differences viewed in the way themes were discussed across participant subgroups, were then reorganized to align with the evaluation questions. Informed by outcome harvesting, teams sought to trace observed consequences and outcomes to initial inputs, including the project’s interventions.

In DRC, observation data were entered into Excel and frequencies of each question were tabulated. Calculations of completeness of DHIS2 aggregate and DHIS2 Tracker data were performed within the DHIS2 system. Similarly in Niger, Parsyl data availability and completeness was assessed within the system. In Vietnam, microplanning indicator data were entered into Excel and frequencies of each indicator were tabulated.

### Ethical considerations

Before participating in interviews or observations, all participants provided verbal informed consent. They were made aware that their participation was voluntary and that any information they shared would be deidentified so that it could not be linked back to them. In DRC, ethical approval for interviews, observations and secondary data analysis was obtained from the Kinshasa School of Public Health (PATH-RDC/CO/TH/KBB/03/02–2023) in March of 2023. The evaluations in Niger and Vietnam were determined to be nonhuman subject research by JSI’s Institutional Review Board (#22-85E) and PATH’s Research Determination Committee (RES-00548), respectively.

## RESULTS

A total of 76 health system actors contributed interview data across the three countries. We observed four data review meetings (two in DRC, two in Niger) and observed data entry and management practices in 11 facilities in DRC (Table 4).

Following the logic of our evaluation questions and TOC, results are presented by evaluation question (EQ), tracing decisions and adaptations (EQ1) to resulting interventions and to implementation and behavior change (EQ2), through to changes in data availability, quality and use (EQ2) and changes to the eHealth building blocks of other health system outcomes (EQ3).

### EQ 1: How were COVID-19 vaccine-related data and digital interventions refined or adapted based on gaps and challenges identified? To what extent, and why or why not, did adaptations consider the countries’ digital health strategies or architecture or leverage existing global goods?

As noted in the background, adaptations in the three countries occurred due to a combination of ideas and evidence about the challenges with the systems and tools in place for COVID-19 vaccine-related data and the availability of financial resources and technical advocacy during the COVID-19 emergency response.

Niger and Vietnam’s decisions moved quickly, supported by technical and financial resources made available through emergency COVID-19 funding. Interviews suggest that the Niger EPI’s decision to introduce Parsyl at national scale was driven by the positive results of the pilot, the donation of devices by Parsyl and the technical assistance provided by the project. In Vietnam, according to respondents, the new tool’s adoption was facilitated not only by the apparent need but because the project involved government stakeholders in the development of the tool.

The decision-making process in DRC took months of discussion and negotiation, described below, seemingly due to the ‘lock-in effects’ of DHIS2 Tracker [28]. Respondents noted that this decision to use DHIS2 Tracker was the result of influence from certain global technical partners and that many other countries had deployed DHIS2 Tracker. As one national stakeholder put it, ‘There was no exploration of tools to see which was the most appropriate for the country…the choice of DHIS2 Tracker was explained by the fact that the tool was used by many of the countries.’ One respondent noted that some stakeholders believed that the problems were largely technical in nature and could be resolved by system improvements, more training or more tablets. The informant explained that others, including USAID in DRC, felt the problems were related to human resources and behavioral factors and necessitated a more creative approach.

In March 2022, the DRC project proposed interventions that would contribute to longer-term systems strengthening by focusing on improving skills and processes to strengthen data validation and use in addition to the short-term need to address missing data. Documents indicate the project and other technical partners began to advocate for a shift towards DHIS2 aggregate to mirror routine immunization workflows, which consisted of facility-level health staff entering vaccination data in paper forms, which were sent to the health zone level for aggregated data entry into DHIS2. To help reach consensus, in September 2022, the project and EPI hosted a cocreation workshop with key stakeholders involved in COVID-19 vaccination data and data systems to discuss data challenges and viable solutions. Key recommendations from this workshop were taken up by EPI and the Division of Health Informatics in October 2022. They included: using DHIS2 aggregate to achieve a more complete picture of COVID-19 vaccination around the country and to better align with existing immunization data workflows; revising the data collection tools used at the operational level, including vaccination registers and tally sheets, to collect vaccination data about specific subpopulations; ensuring the availability of paper forms; improving the training and supervision received by data clerks and managers; and committing to monthly data review meetings. Together these interventions form a holistic package meant to improve the timely availability of quality data; their implementation was supported by the project.

Adaptations in Vietnam and DRC were primarily driven by the poor fit of the original interventions to the country’s digital health enabling environment. Resulting interventions in all three countries focused on addressing user needs and capabilities. In Vietnam, respondents noted the tool was developed to consider existing health worker digital literacy and automated for efficiency. According to respondents, the new microplanning tool was not intended to replace the existing national systems; rather, it was implemented as a temporary measure to fill gaps until the national systems could be updated and improved. While Niger has a national eHealth strategy [29], the existing cold chain monitoring system is largely paper-based as in most low-income countries. During conversations about Parsyl, respondents noted there was limited discussion of whether and how the device and its software aligned with or advanced the existing or planned digital health architecture or the eHealth strategy. Decisions to adopt Parsyl did consider the human resource capacity needed to use the intervention, and the pilot showed that the device was user friendly and relatively easily adopted by users.

### EQ 2: Whether and how did these adaptations or new strategies contribute to improved outcomes related to the availability, quality and use of COVID-19 vaccine-related data for program decision-making?

#### Implementation and uptake of the interventions

The adaptation processes described above resulted in the package of interventions summarized in Table 1. Across all countries, evaluators observed the successful implementation of these interventions and observed or documented through interviews user capabilities, motivation and opportunity to use the interventions [24]. In DRC, evaluators observed that new paper forms were available in facilities but written guidelines or job aids were not. Project records indicated that vaccinators and data clerks had been trained in the new tools and processes. Evaluators observed that personnel at nearly all the sites correctly described or demonstrated key data entry procedures. The DHIS2 aggregate module was operational and respondents had positive perceptions of the DHIS2 aggregate system. One data manager mentioned that DHIS2 aggregate entry was clear and straightforward with the added benefit that everyone could see the data on the platform unlike the Excel database, which was multiple separate files, and in contrast to DHIS2 Tracker for which so much data was missing online.

In Niger, at the time of this evaluation’s data collection in May 2023, RTMDs had been installed on CCE for ~5 months, with ~65% of RTMD devices sending data for that month. SMS alerts of temperature excursions were being sent to target staff and temperature data were available on the Parsyl dashboard, according to a review of these dashboards. The MOH developed a CCE maintenance log register and staff utilized it to document maintenance activities.

In Vietnam, project records showed that the new tool was implemented in the five project provinces and project staff provided training and supportive supervision to its users. As of September 2022, 1485 facilities reported using the new tool. Respondents at all levels of the health system in Vietnam identified time savings and reduced workload as a benefit of the new microplanning tool. At the district and province level, health managers no longer had to rely on receiving the data from lower levels in different formats via different channels, including email or Zalo messaging groups, which necessitated aggregating data from different sources and software and redoing reports for every vaccine batch. Rather, they received data in a standardized format from the districts, allowing for faster decision-making and planning for the next allocation of vaccines. As stated by a provincial health official in Quang Nam Province:

‘Before the project intervention, we relied solely on Gmail and Zalo reports, which were not well organized for updates. Each time we reported, we had to redo the report separately. With the implementation of this tool, once we have investigated and reviewed the updated data and deployed it in batches, it is almost ready for the next deployment. This convenience is beneficial because it ensures precision in the plan.’

At the commune level, it was common to require multiple commune staff to complete the planning process for each vaccination round, whereas respondents reported that the new microplanning tool typically could be updated by one person in less than an hour. District and commune staff identified several secondary effects of this time savings, including less work stress and pressure leading to higher motivation and job satisfaction. Health workers noted that the time savings offered by the new microplanning tool allowed staff time to engage in other work, such as consulting with clients and planning vaccination sessions.

We found that improved capabilities were not universal across respondents and a common theme across countries was the desire for more training. In Vietnam, despite developing an Excel-based tool to avoid digital literacy gaps, most district- and commune-level health workers initially found the tool confusing and difficult to use, and some were reluctant to change their processes. While the majority of health workers became proficient in using the tool after training and continued supportive supervision, some expressed continued confusion and reluctance to use the tool. In Niger, all target users (regional and district immunization officers and CCE technicians) received training from the project and the project had placed at least one staff person in each region to support the implementation of this intervention and provide supportive supervision. However, interviews with health systems staff indicated that additional training was desired for some users to interpret and act on the SMS alerts. In some districts, interviews indicated that district-level staff involved in the CCE system were not aware of how to access the RTMD data or were not able to access it because most had to use their own phone and pay for the cellular data costs, limiting their opportunity and motivation to access and use the data. During the evaluation, the draft standard operating procedures (SOPs) describing the processes for using the data had not yet been fully endorsed or disseminated, leaving staff without key guidelines on how to effectively integrate and use the RMTDs.

#### Unintended consequences related to new tools or parallel tools

In all countries, intervention users noted unintended consequences related to the parallel or concurrent use of more than one intervention. In DRC, many respondents at the health zone level acknowledged that the introduction of a DHIS2 aggregate workflow added to, rather than replaced, the Excel workflow, resulting in decreased motivation. Respondents at operational and national levels, including from within the EPI, questioned why the Excel tool was still being used when the idea of retroactively entering data into DHIS2 aggregate was, in part, to phase out the Excel database in favor of adopting DHIS2 aggregate. National stakeholders attributed its continued use to the fact that the ministry had not yet officially communicated that the Excel database must be retired from use and therefore health zone staff were collecting and storing COVID-19 vaccination data in both Excel and DHIS2 aggregate in order to be compliant with ministry guidance. In Vietnam, some health workers also mentioned the need to use the previous microplans in parallel to the new microplan due to requirements from other departments. For example, some health workers described having to use the Word version of the plan for submission to the District or Commune People’s Committee in addition to the microplan produced by the new tool. Similarly in Niger, the adoption of the RTMD was in the context of the ongoing use of the paper-based temperature monitoring system per WHO guidance; staff remained diligent about collecting daily temperature data and recording them on a paper-based temperature monitoring sheet.

#### Consequences of the interventions on data availability, quality and use

All three countries showed some improvements in data availability, quality and use because of these interventions. In Niger and Vietnam, the interventions were designed to automatically improve the availability of quality data with clear prompts for taking action on the data, whereas in DRC, improved data availability, quality and use required many more human and behavioral inputs. For instance, in Niger interviews and project records indicated that target staff with access to the Parsyl dashboards and SMS reminders could see real-time temperature data. When target staff receives an SMS alert they should investigate the alert, take action to address it and document their action in the Parsyl app. During this early implementation period, there were 33 distinct instances of actions documented on the Parsyl dashboard in the evaluation regions. The actions included transferring vaccines to other CCE, informing supervisors of the alert and adjusting the thermostat. At the regional level, we observed that two of the three intervention regions were reporting data from the RTMDs in their monthly logistics reports. During the evaluation, we did not document organic use or interpretation of temperature data in these regional or national meetings, suggesting that the longer-term objective of building the culture, skills and processes for temperature data use requires more work.

**Table 5:** Comparison of microplan data quality and availability benchmarks, preintervention and postintervention in Vietnam

**Quality benchmark**		**Preintervention, *n* (%)**	**Postintervention, *n* (%)**
	Does the plan specify population numbers by target group?	6 (27)	22 (100)
	Does the plan specify the capacity to store vaccines at the facility?	1 (5)	21 (95)
	Does the plan clearly mention the facility’s capacity in terms of human resource to participate in the COVID-19 vaccination campaign?	12 (55)	22 (100)
	Does the plan provide an estimate of the needs for each vaccine type by age group and immunization schedule for each group?	5 (23)	22 (100)
**Master plan for the campaign**	Does the plan provide an estimate of each consumable needed for the vaccines by each target group?	4 (18)	22 (100)
	Does the plan specify the quantity of each vaccine to be received?	12 (55)	22 (100)
	Does the plan specify the group to be given priority for that vaccination session?	19 (86)	22 (100)
	Does the plan clearly specify the site and the strategy of vaccination (at the health center or vaccination points elsewhere) for each batch of vaccine received?	18 (82)	19 (86)
**Microplan for each vaccination session**	Does the plan specifically assign staff in the immunization line?	12 (55)	18 (82)

In Vietnam, analysis of the preintervention and postintervention microplans showed that postintervention microplans increased data availability and quality by an average of 49% across data completeness and availability benchmarks (Table 5). The new microplanning tool featured embedded automatic calculations, which obviated the need for any manual calculations and aggregation that had previously caused frequent data errors. Respondents reported that plans more accurately reflected the demographics of individuals left to be vaccinated due to inclusion of more specific target groups in the tool. Additionally, health workers described an improved ability to plan for sessions based on the detailed information built into the microplanning tool, including estimates of the number of health workers needed to carry out vaccination sessions and their specific roles; estimates of cold chain capacity needed based on each brand of vaccine; and estimates of other consumables needed, reducing wastage.

Although respondents in Vietnam described positive impacts of more detailed and accurate data on the vaccine allocation process, the qualitative evidence was mixed as to whether the implementation of the new microplanning tool had an impact on COVID-19 vaccination coverage. According to provincial-level health workers and some district-level health workers, the tool was an effective mechanism for managing and estimating vaccination targets but stopped short at having an impact on the number of people vaccinated. In contrast, some district- and commune-level health workers explained that being able to quickly calculate specific information on the remaining subjects to be vaccinated allowed health workers more time to engage in proactive demand creation to make sure that clients attended the vaccination session, thereby increasing the number of people vaccinated overall.

In DRC, analysis of secondary data suggests that the interventions contributed to improved completeness of vaccination data, which makes more data available to use. Informants generally perceived the transition to DHIS2 aggregate to be responsible for increased data completeness and praised the accessibility of its data. Data completeness was frequently described by informants in quantitative terms such as moving from ‘around 40%...[up to] about 78%.’ Secondary analysis of the volume of data recorded in the two health zones illustrated that, as of June 2023, the DHIS2 aggregate database had recorded 76 978 doses of COVID-19 vaccine administered while DHIS2 Tracker had recorded 45 340. This suggests that the DHIS2 aggregate database reflected a greater number of actual doses administered and therefore contained more complete data than DHIS2 Tracker. Respondents at the health zone and national levels spoke of the improved disaggregation of the data in DHIS2 aggregate, as compared to the Excel database, due to the revised paper forms. One data manager said the data reported in DHIS2 aggregate are now more accurate but didn’t explain in what way. A national stakeholder noted that this adaptation allowed the EPI to track the vaccination of special groups, such as those over the age of 55 years, internally displaced persons and those with comorbidities.

In DRC, respondents routinely spoke of *using* data to refer to analyzing or verifying the quality of data but not making programmatic decisions based on the data. Conversations about data quality and data use usually revolved around checking, verifying and discussing data with the end goal of improving data quality. For example, informants at the antenna level spoke of following up with staff at the zonal level and informants at the zonal level spoke of following up with staff at the site level. At the national level, one stakeholder noted the importance of data quality and how it was crucial for accurate tracking of vaccinated individuals. Many respondents spoke about providing feedback about data incoherencies during zonal review meetings. One data manager noted that previously, COVID-19 vaccination data were not analyzed and discussed during zonal monitoring meetings in the health zones, but following the recommendations of the EPI and the project in February 2023, the health zones were discussing all data, including COVID-19 vaccination data. The evaluators were unable to confirm this change during observation of zonal review meetings since the health zone staff responsible for discussing COVID-19 data were not present at the two observed meetings in May 2023. Data visualization was only mentioned insofar as DHIS2’s capacity to produce dashboards; however, during observation of zonal data review meetings, the evaluators only observed tables of raw health facility data.

### EQ3: Whether and how did these digital health adaptations or new strategies strengthen the digital health enabling environment across the eHealth building blocks in evaluation countries or the broader health or immunization system? What were the barriers and facilitators to strengthening the digital health enabling environment or health systems through these investments?

Across the three countries, we found little evidence of strengthened eHealth building blocks as a result of these interventions. No respondents organically mentioned consequences related to the eHealth building blocks when asked to describe overall results of the interventions. When probed, one national respondent in DRC said it may be too early to tell but they hoped the experience would be a reminder that new uses of digital global goods (such as DHIS2 Tracker for vaccination) must be adapted for each country’s reality. Project records and interviews from Niger and Vietnam indicated that strengthening the eHealth building blocks was not the intention of these interventions. In Niger, interviews with the project team noted that the RTMD intervention was not initially designed to strengthen the eHealth building blocks, in part due to the lack of awareness about opportunities to do so and/or the lack of guidance on aligning COVID-19 data and digital investments with Niger’s eHealth building blocks. In Vietnam, the microplanning tool was designed as a stopgap solution to equip health workers with a planning tool while the national COVID-19 vaccination software could be updated. Qualitative evidence suggests that the introduction of the new microplanning tool has had limited impact on the overall eHealth enabling environment in Vietnam.

Although respondents did not mention positive consequences related to human resource capacity for digital health, site-level observation (DRC) and interviews (Niger, Vietnam) concluded that users had the capabilities to use the data forms and systems, which is an improvement compared to prior assessments (DRC, Vietnam) or the preintervention situation (Niger).

In DRC, broader leadership and governance concerns were raised with regards to the way in which coordination of COVID-19 vaccination funds were originally housed outside the EPI. One informant implied that the effort to transition the coordinating body under the EPI came too late and the lack of EPI control over COVID-19 vaccination coordination, combined with the fact that COVID-19 funding was dwindling, demotivated national stakeholders and their willingness to take ownership over COVID-19 vaccination data system improvements. An outcome of this cited by one respondent was the lack of a path to transition away from the Excel database.

#### Consequences on broader immunization or systems strengthening

In Niger, the RTMD intervention, by design, should strengthen the cold chain system for all vaccines. Interviews indicated the EPI desires the intervention be extended to the health facility level across the country. In DRC, respondents spoke of positive consequences of the longer-term immunization and data use outcomes, including the spillover effects on the routinization or integration of COVID-19 vaccination data systems and workflows with other routine health service workflows. One respondent noted this helps with goals to integrate COVID-19 vaccination with other health services. In Vietnam, although many health workers expressed the desire to apply the tool for planning for other vaccines and interventions, based on its effectiveness and time savings for staff, they also acknowledged that the existing national immunization information system routine immunization microplanning tool functions well, and there are no plans for adoption of the new microplanning tool at the national level. In the province of Quang Nam, the tool was partially used for planning Japanese encephalitis vaccination catch-up campaigns but only in a limited capacity. Regional stakeholders emphasized that any effort to integrate the tool into the national eHealth infrastructure would necessitate strong MOH buy-in and careful consideration of the potential value add.

## DISCUSSION

Across the three countries, the types of adaptations and the reasons behind them differed. All three cases reflect the accelerated timelines of deploying and adopting DHIs in emergency contexts and the importance of being able to adapt in real time. In Vietnam, challenges with the initial microplanning tool resulted in a short-term but effective adaptation. In Niger, challenges with the status quo combined with the visibility of the COVID-19 vaccine cold chain led to a promising DHI deployment but without clear plans for long-term funding. In DRC, the visibility of incomplete data led to a larger system transformation to improve alignment and sustainability of national digital health systems but challenges remain. These solutions rose to the top because they addressed visible problems and were deemed feasible, particularly as it related to human resource capacity.

All three countries demonstrated the importance of investing in holistic packages of interventions that addressed user capabilities, motivations and opportunities to use data, in line with existing research [2]. First attempts at rolling out COVID-19 vaccine data-related interventions illustrated that DHIs alone cannot achieve intended programmatic outcomes. Accordingly, the TOC for this supplement includes a range of interventions that target all aspects of user and organizational skills, processes, incentives and the broader digital health enabling environment [22].

However, the extent to which these adaptations considered the countries’ digital health strategies or architecture, or leverage global goods, varied. In Vietnam, the national architecture and system was itself the challenge, but the decision to create a stopgap solution was made with explicit awareness of the national digital health strategy, particularly that the solution should not replace the national system in the long term. In DRC, the original intervention (DHIS2 Tracker) as well as its adaptations (DHIS2 aggregate) aimed to explicitly align with national architecture and global goods (DHIS2); however, considerations of human and financial resources tipped the balance towards DHIS2 aggregate more than other factors. DHIS2 Tracker may be an example of a global good that aligned with national strategies and architecture but was not accompanied with adequate change management for success. This misalignment was likely exacerbated in the emergency context.

In Niger, the RTMD intervention represented the digitalization of CCE monitoring but this adaptation did not extensively consider existing architecture or global goods. We expect this digital adaptation was possible because of the emergency context, and yet the urgency of the COVID-19 vaccine cold chain needs may have limited the ability of stakeholders to take the time to consider a longer-term roadmap for digitizing all of Niger’s vaccine logistics management systems and planning for interoperability and alignment of RTMDs with planned global goods such as electronic logistics management information systems. We found that, except for conversations in DRC, the project teams did not initially have explicit guidance from USAID on how data and digital investments might be designed to contribute to broader eHealth goals. As a result, counties’ digital health strategies, architecture or existing global goods were not always considered systematically and directly in the interventions proposed or accepted.

Prepandemic investments in the eHealth enabling environment in countries such as Vietnam allowed it to quickly identify and act on challenges. In such countries, with strong eHealth leadership and governance, time-limited gap-filling solutions may be appropriate to address specific challenges during an emergency provided there is a clear strategy for their use and eventual decommission, as ‘temporary measures have a nasty habit of outlasting emergencies’ [30]. Countries like Vietnam may also be positioned to use emergency funding catalytically towards eHealth strategic goals. In countries with more nascent digital health maturity, time and resources were likely wasted on deploying unsuitable interventions. These countries and their technical partners likely need more support in considering how to align with existing architecture, software and human resources and how DHI investments can contribute towards even nascent digital health strategies.

### Outcomes

Improvements occurred along the evaluation TOC but not consistently. Assessment of capabilities, opportunity and motivation [24] to use the interventions helps explain the implementation and resulting consequences on immediate and intermediate outcomes. For example, all interventions were designed with human resource capabilities in mind and the broader intervention packages sought to address opportunities to use the interventions through change management strategies. Across the countries, the project teams worked closely with government stakeholders and other key influencers to ensure acceptance and support for the interventions. Critically, and as described elsewhere [2], built-in functionality to make data directly available to users and nudge actions contributed to data use. For example, the RTMDs in Niger sent SMS alerts of cold chain excursions to individual users. In a single step, this ‘push’ mechanism makes data available and interprets the data for the user, unlike in DRC where the user still had to take an action to log in, look at a dashboard or report and interpret the data. Similarly, the microplanning tool in Vietnam was designed to automatically make key data available to users in a way that directly linked to its use for planning. In Niger and DRC, we found that individual and organization capabilities, opportunities and motivation to use data could still be improved in addition to change management. The issuance of SOPs may help, but in both countries more investment is needed to strengthen the culture of taking action on data.

Considering these interventions were designed first and foremost to help achieve vaccination goals, we found limited evidence that they strengthened the eHealth building blocks during this short period of time, as was proposed might happen in the TOC. There is some evidence that these interventions strengthened the health workforce’s digital health capacity by including high-quality training and supportive supervision on the digital tools. It may be too early to measure whether in DRC the efforts to coordinate stakeholders to align with realistic national digital health architecture and goals have contributed to strengthening building blocks related to coordination and to leadership and governance. A barrier that emerged in interviews was that of the dual coordinating structures for COVID-19 vaccine-related data: the EPI program and the national COVID-19 coordinating committee. Most countries developed a coordinating structure outside of the EPI to accelerate COVID-19-related decision-making but this poses longer-term sustainability challenges for the decisions that were made by those bodies.

In DRC and Niger, we found some evidence that the interventions are contributing to broader immunization and health systems goals. In DRC, the project designed the DHIS2 aggregate-related interventions to align with existing tools, systems and workflows for routine immunization and other health services, which is now helpful as the country integrates COVID-19 vaccination into other routine health services. Niger’s RTMD intervention affects all of the vaccine cold chain, not just COVID-19 vaccines. As an immunization-focused project, it was natural that it aimed to strengthen broader immunization systems when designing COVID-19 vaccine-related interventions.

### Limitations

This evaluation had several limitations. First, interventions were implemented for different periods of time in the evaluation countries; particularly in Niger, these findings represent only 5 months of implementation. Yet, we believe the observations offer important insights for continuous improvement or adaptive management. It was not possible to systematically measure longer-term outcomes related to possible broader consequences to national information or immunization systems. It was not possible to design an experimental or quasi-experimental study in this real-world setting, but our integration of theory- and complexity-aware evaluation approaches strengthens the rigor. In retrospect, we questioned in DRC and Niger whether we interviewed enough respondents as their opinions varied across levels of the health system in unexpected ways. Finally, members of the evaluation team also played a role in intervention design. While we prioritized reflexivity during the analysis process—the process in qualitative research of acknowledging one’s role and biases in the research process—it is possible that respondents exhibited social desirability bias or that the evaluation team’s perspectives colored the data analysis.

## CONCLUSION

The COVID-19 pandemic spurred immense innovation in all areas of life. Similar to the West African Ebola outbreak, the combination of need and resources contributed to the rapid deployment of new DHIs and the rapid adoption of existing DHIs [31]. In the case of COVID-19 vaccination data and digital interventions, many were deployed or adopted without adequate consideration of existing architecture, resources and change management required. Many of these interventions persist without producing expected benefits. We highlight three countries where adaptation occurred with largely positive results related to data availability, quality and use, largely because the adapted interventions reflected good practices related to the importance of holistic interventions and change management strategies during digital transformation [2, 5]. While it is too early to tell if the interventions in DRC and Niger will lead to longer-term benefits for information or immunization systems, we note that these benefits are only likely to occur if they are planned from the start. Funders and implementers of DHIs should consider practical steps to support or possibly strengthen country eHealth enabling environments though DHI investments in emergency contexts.

## STUDY FUNDING

MOMENTUM Routine Immunization Transformation and Equity and this research was funded by the U.S. Agency for International Development (USAID) as part of the MOMENTUM suite of awards and implemented by the JSI Research & Training Institute, Inc. with partners PATH, Accenture Development Partnerships, Results for Development, CORE Group, and The Manoff Group under USAID cooperative agreement #7200AA20CA00017. For more information about MOMENTUM, visit www.usaidmomentum.org. The contents of this journal article are the sole responsibility of the JSI Research & Training Institute, Inc. and do not necessarily reflect the views of USAID or the United States Government.

## CONFLICT OF INTEREST

The authors declare no conflicts of interest.

## AUTHORS’ CONTRIBUTIONS

G.M. (Formal analysis [Equal], Investigation [Equal], Methodology [Equal]), C.K. (Formal analysis [Equal], Investigation [Equal], Methodology [Equal], Writing—review and editing [Equal]), J.N. (Data curation [Equal], Formal analysis [Equal], Investigation [Equal]), E.P. (Formal analysis [Equal], Investigation [Equal]), P.M. (Formal analysis [Equal], Investigation [Equal]), D.C. (Supervision [Equal]), S.D. (Data curation [Equal], Investigation [Equal], Methodology [Equal]), D.C. (Investigation [Equal], Methodology [Equal]), T.P. (Data curation [Equal], Investigation [Equal], Methodology [Equal]), T.L. (Data curation [Equal], Investigation [Equal], Methodology [Equal]), H.I. (Data curation [Equal], Investigation [Equal]), Y.A. (Writing—review and editing [Equal]), W.P. (Formal analysis [Equal], Investigation [Equal], Methodology [Equal], Project administration [Equal], Writing—original draft [Equal], Writing—review and editing [Equal]), A.C. (Data curation [Equal], Formal analysis [Equal], Investigation [Equal], Methodology [Equal], Writing—original draft [Equal], Writing—review and editing [Equal]), A.H. (Data curation [Equal], Formal analysis [Equal]), E.E. (Data curation [Equal], Formal analysis [Equal], Investigation [Equal], Methodology [Equal], Project administration [Equal], Writing—original draft [Equal], Writing—review and editing [Equal]), G.A. (Data curation [Equal], Formal analysis [Equal], Investigation [Equal], Methodology [Equal], Project administration [Equal], Writing—original draft [Equal], Writing—review and editing [Equal]), and J.S. (Conceptualization [Equal], Funding acquisition [Equal], Methodology [Equal], Supervision [Equal], Writing—original draft [Equal], Writing—review and editing [Equal]).

## DATA AVAILABILITY

Deidentified study data from project documents, interviews, secondary data and observation notes are available upon request from the corresponding author.
